# Innovative Strategies for Drug Delivery to the Ocular Posterior Segment

**DOI:** 10.3390/pharmaceutics15071862

**Published:** 2023-07-01

**Authors:** Andrea Gabai, Marco Zeppieri, Lucia Finocchio, Carlo Salati

**Affiliations:** 1 Department of Ophthalmology, University Hospital of Udine, 33100 Udine, Italy; 2 Department of Ophthalmology, Nuovo Ospedale Santo Stefano, 59100 Prato, Italy

**Keywords:** drug delivery systems (DDSs), nanotechnology, matrices, ocular posterior segment, vitreoretinal

## Abstract

Innovative and new drug delivery systems (DDSs) have recently been developed to vehicle treatments and drugs to the ocular posterior segment and the retina. New formulations and technological developments, such as nanotechnology, novel matrices, and non-traditional treatment strategies, open new perspectives in this field. The aim of this mini-review is to highlight promising strategies reported in the current literature based on innovative routes to overcome the anatomical and physiological barriers of the vitreoretinal structures. The paper also describes the challenges in finding appropriate and pertinent treatments that provide safety and efficacy and the problems related to patient compliance, acceptability, effectiveness, and sustained drug delivery. The clinical application of these experimental approaches can help pave the way for standardizing the use of DDSs in developing enhanced treatment strategies and personalized therapeutic options for ocular pathologies.

## 1. Introduction

New drug delivery methods to target specific ocular tissues and treat debilitating ocular diseases have been proposed in the past decade. Of all ocular diseases, 55% originate from the posterior segment [1]. Retinal diseases, such as age-related macular degeneration (AMD), diabetic retinopathy (DR), diabetic macular edema (DME), endophthalmitis, viral retinitis, proliferative vitreoretinopathy (PVR), posterior uveitis, retinal vascular occlusions, retinitis pigmentosa (RP), and inherited retinal diseases (IRDs), represent the leading cause of vision impairment [2,3].

**Figure 1:**
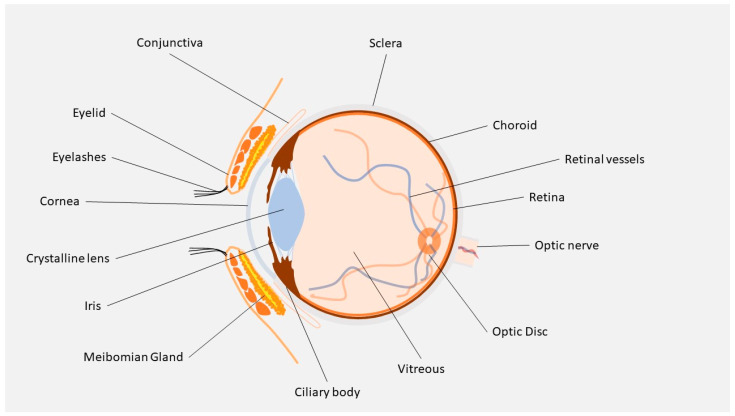
Ocular anatomy.

The presence of static barriers (different layers of the cornea, sclera, retina, blood–aqueous (BAB) and (BRB) blood–retinal barriers) (Figure 1) and dynamic barriers (conjunctival and choroidal blood flow, tear turnover, and lymphatic clearance) represent an obstacle to the delivery of a drug to a particular ocular tissue and to treating these retinal diseases [4]. The research field based on ocular “drug delivery”, which refers to numerous innovative techniques to target retinal tissue and overcome ocular barriers, has witnessed huge growth in recent years.

Depending on disease type, drug property, and target site, drugs can be administered through different routes, including topical, intravitreal, periocular, and systemic. Several types of ophthalmic drug delivery systems (DDSs) are currently available on the market, and range from eyedrops, eye ointments, gels, and ocular inserts, such as eye dosage formulations that are created to increase the holding time of drugs in the eye. Topical drug instillation on the ocular surface is a common and non-invasive application method for the treatment modalities of eye disorders. This route, however, offers suboptimal ocular bioavailability. Studies have shown that 90% of eyedrops available on the market only provide 5% of drug bioavailability, while the rest of the drug gets washed away through different elimination routes, such as tear fluid, nasolacrimal secretion, protein binding, enzymatic degradation, or metabolism by protease and esterase enzyme [5]. Despite the limits of topical treatments, current studies have reported promising results for substances delivered to the retina, which suggests that topical treatment of retinal diseases might be possible in the future [6,7].

Injection into the globe is very often recommended for drug delivery into the posterior region, but it is painful, causes patient non-compliance, has risks of infection, and is associated with various side effects. Over the past decade, thanks to advances in nanotechnology and the development of many drug delivery systems, such as implants, in situ gel, contact lenses, microneedles, liposomes, nanomicelles, dendrimers, and other nanoparticles (NPs), numerous drug-delivery solutions have been proposed. The polymeric nature of these various technologies includes the distinction between microparticles, which are polymeric systems larger than 1 µm, and nanoparticles that have a smaller diameter. Structurally, these particles can be broadly described as micro/nanospheres, when the loaded drug is integrated in the matrix and nano/microcapsules, when the drug is enclosed in a polymeric shell. Although most of the studies in this field are still in the preclinical stages, micro/nanotechnology-based DDSs possess great potential to solve the shortcomings of currently available drug delivery systems. These new strategies can potentially allow safe and effective drug administration to the ocular posterior segment and retina [8,9,10,11].

In this mini-review, we attempt to focus on and highlight promising strategies reported in the current literature based on innovative routes to overcome the anatomical and physiological barriers of the vitreoretinal structures. We also describe the challenges in finding appropriate and pertinent treatments for delivering therapeutic agents to retinal tissues in a safe and effective manner.

## 2. Ocular Barriers

Pharmaceutical formulations via the anterior segment, mostly liquid solutions, or suspensions, are the most common treatment used in ophthalmology. These locally administered drops include a broad range of molecules, such as antibiotics, anti-inflammatory agents, anti-glaucomatous drops, lubricants, and diagnostic agents. Topical formulations tend to be used for the ocular surface and do not allow topical drugs to reach therapeutic concentrations in the posterior ocular segment (vitreous, retina, choroid) due to several anatomical and functional barriers.

### 2.1. Lacrimal Wash-Out and Mucosal Capillary Circulation

The first and most important factor that decreases topic drop efficacy in the posterior segment is the continuous lacrimal wash-out of the ocular surface. This shortens the contact between eyedrops and the ocular surface so that an instilled volume of topical drug is usually drained via the nasolacrimal duct in approximately 2 min [12]. Capillary vessels, both in the nasal mucosa and conjunctiva, also prevent a portion of the active drug from reaching therapeutic concentration within the eye by dispersing molecules in the systemic blood flow [13].

### 2.2. Cornea

The epithelium of the cornea is the principal barrier of this multilayered bio-membrane. Epithelial cells are lipophilic, tightly interconnected, organized in a 3 to 6 layer structure, and continuously replaced with newer cells migrating from the limbus to the corneal apex. The intercellular tight junctions limit the paracellular passage of hydrophilic molecules and ions, making the cornea much more permeable to lipophilic drugs, and capable of transcellular passage. The corneal stroma, which is rich in collagen fibrils and glycosaminoglycan, is hydrophilic, thus limiting the absorption rate of lipophilic molecules crossing the epithelium. The endothelium, with its monolayer structure, seems to offer little resistance to lipophilic substances. For these reasons, corneal permeation is currently the preferred way to deliver lipophilic drugs with a small molecular weight in the anterior chamber [14,15].

### 2.3. Conjunctiva

Studies have reported that the conjunctiva is approximately 20 times more permeable than the cornea, and that allows for the absorption of more hydrophilic and larger molecules, thus offering a potential route of administration for new therapeutic proteins and peptides [16,17,18].

### 2.4. Sclera

The sclera is composed of collagen fibers embedded in mucopolysaccharides, thus making it more permeable to drugs than the cornea and less than the conjunctiva. It is important to remember that the conjunctiva is the most vascularized and porous structure of the ocular surface [19]. Scleral permeation mostly occurs through passive diffusion, with a permeability coefficient inversely related to the molecular weight of the permeating substances [16,20].

### 2.5. BAB and BRB

Systemic drug administration provides low ocular bioavailability (<5%) due to the limited fraction of the entire blood reaching the eye and the presence of the BAB and BRB, which render systemic administration effective only for drugs with high therapeutic index, and for treatments given in high doses, such as antibiotics and antiviral agents [13,21].

Non-pigmented ciliary cells and endothelial cells of the irideal vessels are the main elements of the BAB. The tight intersections of this structure render the wall of the capillaries impermeable to the plasma albumin, thus avoiding access to the aqueous humor and limiting the passage of hydrophilic drugs from the systemic circulation to the anterior chamber. The disruption of BAB in inflammatory conditions can allow systemic drugs to freely distribute to the anterior chamber [13].

The blood flow through the choroidal vasculature passively contributes to the clearance of systemic drugs, preventing penetration into the eye. The choroidal vessel walls, however, are rather permeable, so blood and drugs can easily leak through. A more active barrier to penetration is represented by the retinal pigment epithelium (RPE) and Bruch’s membrane, which constitute the outer part of the BRB. Retinal vessel endothelium and intertwining connections also block drug passage from systemic circulation to the retina and vitreous, constituting the inner part of the BRB [13,22,23]. The metabolic, molecular, and pharmacokinetic characteristics of blood–ocular barriers are not completely known; future histological and molecular pathologic studies are needed to better understand the underlying mechanisms involved in these structures.

## 3. Methods

We conducted a search of the literature published between 1 January 2002 and 28 February 2023 using MEDLINE (PubMed). The database was first searched using the key words “drug delivery system, ocular posterior segment, AND vitreoretinal, AND nanotechnologies AND matrices”. We considered only studies in English and those with an abstract. The reference lists of all retrieved articles were assessed to identify additional relevant studies. The research of articles was performed using PubMed (https://pubmed.ncbi.nlm.nih.gov, accessed on 28 February 2023).

Only articles with an abstract were considered. Studies in which small case series were described and those that assessed surgical techniques were analyzed. Each study was independently assessed by at least two reviewers (A.G., L.F., and M.Z.), and rating decisions were based on the consensus of the reviewing authors. A total of 233 references were included in the review. This search strategy was limiting in the midst of the vast literature, which could have thus potentially and unintentionally excluded opinion leaders in this field of research.

## 4. Innovative Drug Delivery Systems

### 4.1. Nanomedicine

Systems and devices developed for scientific purposes, with a size scale of cellular and molecular structures ranging from 1 to 1000 nanometer (1 nanometer = 10^−9^ m), are commonly defined as nanotechnology. Nanoparticles (NPs) possess ideal characteristics for ocular drug delivery due to their nanometric and controllable size, and their ability to protect the carried active molecule from degradation, allowing it to be released in a targeted manner and at a controlled rate [24]. In addition, the NP surface area is highly specific and mucoadhesive in comparison to larger bulk polymers due to its high interface availability for hydrogen and ionic bonding, or hydrophobic interaction with mucous surfaces [25]. Drug-loaded NPs with sizes between 50 and 500 nm are the most adapted for ocular delivery, overcoming physiological barriers and mucin mesh [26,27,28]. NPs larger than 1000 nm, however, are unfit to pass through mucous channels, poorly adhere to mucus surfaces, and are easily cleared from them. Nanomaterials studied for retinal diseases consist of amphiphilic molecules, such as liposomes and micelles, polymers, such as dendrimers, and organic and non-organic nanoparticles. The properties of these NPs play a crucial role in their effectiveness in ocular drug delivery. The high surface-to-volume ratio makes them ideal vehicles for therapeutic and targeting agents. Their size must be large enough to avoid drug leakage and dispersion into the blood vessels, and small enough to permit its penetration across the ocular barriers and/or allow for intraocular injection and permanence, when needed.

#### 4.1.1. Liposomes

Liposomes are spherical vesicular structures comprised of amphiphilic molecules, such as phospholipids and sterols, organized to form a lipidic bilayer membrane enclosing an inner aqueous space. Smaller unilamellar liposomes are composed of a bilayer membrane, typically up to 100 nm. Larger versions can have a diameter > 100 nm. Multilamellar liposomimial vesicles, composed of several bilayers, tend to be larger than 500 nm. This architecture, resembling a cellular membrane, provides good biocompatibility and the capability of liposomes to carry considerable quantities of both hydrophobic and hydrophilic drugs and to present variable surface polymers to target different cells and tissues [29].

Liposome-based drug delivery has been studied since 1960 and was the first medical nanotechnology to receive Food and Drugs Administration (FDA) approval in 1995. Verteporfin (Visudyne, Bausch, and Lomb), used to treat choroidal neovascularization (CNV) secondary to AMD, was one of the first commercially available products to adopt liposomes. Choroidal neovascularization was also experimentally treated with liposomes via intravitreal injection, embedding polyethilen glycol (PEG) in the liposomial wall, and via intravenous injection of cationic liposomes loaded with paclitaxel [30,31,32]. Liposomes loaded with the immunosuppressant tacrolimus have been injected into the vitreous to treat autoimmune uveoretinitis. Topical instillation of eyedrops containing TA-loaded liposomes has been shown to improve refractory macular edema both anatomically and functionally [33].

In a study conducted by Gu et al., dexamethasone was successfully delivered in the posterior ocular segment using liposomes. The authors obtained a therapeutic concentration of this drug in the choroid and retina within 2 h from instillation of eyedrops containing dexamethasone salt-loaded liposomes [34]. Despite these promising results, liposome-based ocular therapies present critical drawbacks. NPs cause allergic and hypersensitivity reactions in animal models [35]. Cationic liposomes, in particular, seem more at risk of triggering inflammatory responses [36]. Moreover, liposome aggregation can occur as a consequence of their instability during storage and transportation, resulting in blurred vision with poor functional outcomes, and the need for excipients to stabilize and preserve them limits their therapeutic employment [37,38].

#### 4.1.2. Nanomicelles

Nanomicelles are spherical nanostructures with a hydrophobic stiff core, ideal to encapsulate hydrophobic drugs, and an hydrophilic outer surface facing the aqueous medium. They are usually formulated of a small size (5–30 nm), which makes them ideal for delivering their pharmaceutical content to subcellular targets. Micelles are structurally stable but susceptible to environmental changes (pH, temperature, ionic strength) that can easily precipitate their content (i.e., during storage). Standard, reverse, or unimolecular micelles can be created depending on the amphiphilic molecule and solvent we choose. Furthermore, these NPs have a prolonged circulation time, since they are not recognized by hepatic macrophages, and present a low tendency to aggregate [39,40].

Anti-viral prodrugs were successfully carried to the retina in vivo, using micelles of polyoxyethylene hydroigenated castor oil, across the ocular barriers, after topical administration [41]. Nanomicelles resulting from the polymerization of octoxynol, vitamin E, and tocopherol-PEG-succinate have been used topically to deliver rapamycin to the choroid and retina in vivo, avoiding undesirable concentrations of this antiblastic in the vitreous and with limited cytotoxicity in vitro [42].

Among the hydrophobic drugs that can be vehiculated inside nanomicelles to treat posterior ocular pathologies, we found cyclosporine-A, cyclosporine, and curcumin [43,44,45]. Conjunctival/scleral injection seems a better way to deliver drugs than intraocular injection. Following the conjunctival/scleral route, micelles diffuse through the scleral water channels and then reach the retina without retinal damage after multiple injections [46,47]. Micellar core cross-linking using functional groups and lowering the critical solution temperature have been shown to improve the duration and solubility of nanomicellar preparations [48]. Intravenous administration of nanomicelles was also investigated by Ideta et al. Polyion nanomicelles, encapsulating fluorescein istiocyanate-labeled poly-L-lysine, accumulated around CNV in a rat model of exudative AMD for as long as 168 h after intravenous injection. However, signs of toxicity were detected, demanding further studies on nanomicelle characteristics and administration pathways [49].

Nanotubes can be considered a particular type of nanomicelle. Panda et al. reported the successful intraocular delivery of pazopanib using dipeptidic phenylalanine-alpha and beta-hydroxyphenilalalnine nanotubes as carriers. The preparation was administered via intravitreal injection, obtaining sustained pazopanib concentration in the vitreous, retina, RPE, and choroid for 15 days [50].

#### 4.1.3. Nanospheres and Solid Lipids

Nanospheres are particles encapsulated in polymeric delivery systems. They self-assemble with a uniform distribution of their content, which reduces damage and inflammation in their target tissues. This differentiates them from nanocapsules, which are similar but less uniform polymeric structures [35]. Nanospheres with diameters varying from 50 to 200 nm have been successfully tested in rabbits [51].

Solid lipids are another nanotechnology used to deliver drugs and genes to the retina. As suggested by their name, these particles consist of a solid lipid core stabilized with surfactants. This simple structure makes them resistant, long-standing, and even resistant to autoclave sterilization, but biodegradable and economical to produce, not requiring solvents. Solid lipids have been employed to deliver non-viral gene vectors to RPE cells and photoreceptors in a murine model of X-linked juvenile retinoschisis (XLRS) [52].

#### 4.1.4. Dendrimers

Dendrimers are ramified polymers characterized by a symmetrical core and the presence of functional groups on each branch, with neutral, negative, or positive activity [53,54]. The size usually ranges from 1 to 100 nm, depending on their complexity. PAMAM dendrimers have been used to deliver DEX in vivo, both with topical and subconjunctival administration, in diabetic retinopathy (DR) model rats [55]. Intravitreal and intravenous administration in vivo also showed anti-inflammatory action in murine models by targeting retinal microglia and macrophages, offering interesting perspectives for the treatment of retinopathies where inflammation is dysregulated, such as AMD, DR, and retinal vein occlusion [56]. Neuroinflammation was reduced and photoreceptor loss was halted for 4 weeks in retinal degeneration mice using intravitreal PAMAM dendrimers carrying steroids [57].

Dendrimer clearance was seen to be slowed in target tissues, where it is more needed, than in non-target organs, such as the healthy eye, and interestingly, in comparison to the half-life of other drugs, such as bevacizumab [58].

Dendrimers loaded with brimonidine-tartrate were demonstrated to be more effective than the ordinary topical formulation of this drug in glaucomatous model rats [59]. Similarly, IOP reduction was observed with acetazolamide-loaded dendrimers in albino rabbits [60]. Mouse models of retinoblastoma responded well to PAMAM dendrimers loaded with carboplatin, which, after subconjunctival injection and scleral penetration, reached the tumor, suppressing its vasculature for 22 days [61].

Considerable concerns remain about dendrimers’ safety due to their cytotoxicity. Since the main mechanism for their toxic effect seems to be the disruptive interaction between the positive charge on the dendrimer and the negatively charged biological membranes, several strategies to impede this interaction have been studied, such as acetylation, PEGylation, and peptide conjugation [62].

#### 4.1.5. Organic Nanopolymers

Several types of biodegradable polymers have been developed to deliver ocular drugs in a safe, effective, and sustained way. Among these chemical compounds, we found polyvinylpyrrolidone (PVP), polylactic acid (PLA), polyglycolide (PGA), and their copolymers poly(lactic-co-glycolic acid) (PLGA), poly(n-butyl cyanoacrylate) (PBCA), and polycaprolactone (PCL) [22].

PVP can be injected in hydrogel form, presenting stable retention at the injection site, lasting several weeks. Polymers of PVP have been tested as artificial vitreous substances (cross-linked PVP), lens regeneration scaffolds, and slow-release implants for anti-glaucomatous drugs, acting for 300 days [63,64,65,66]. Small fluorescent hydrophobic molecules conjugated to PVP were effectively delivered to retinal cells after intravenous administration in rats by Tawfik et al. [67]. These results indicate that PVP nanoparticles are promising carriers for hydrophobic drugs in the retina. Nevertheless, other studies have shown possible side effects of this compound, such as corneal and vitreous opacification and inflammation, suggesting that further investigations are required to ameliorate this delivery strategy [68].

PLGA, a well-established and FDA-approved polymer used for the preparation of drug-delivering nanoparticles due to its biodegradability, is the most used drug delivery polymer in ophthalmology. Innovative uses of PLGA to treat retinal disorders have been reported by several authors. PLGA NPs loaded with dexamethasone and injected into the vitreous were able to sustain 50% of the drug level for one month, as compared to the 7 days obtained injecting a simple dexamethasone solution [69]. Light-responsive implants containing PLGA NPs were injected in situ to deliver peptides to the posterior segment [70]. Fragments of plasminogen with anti-angiogenic effect (K5) were delivered to the retina, per intravitreal injection, loaded in PLGA-chitosan nanoparticles. Expressed K5 was detectable in the murine model retina more than 2 weeks after the injection, whereas inflammation resolved in 3 days [71]. Transferrin conjugation to PLGA NPs allowed precise delivery via intravenous injection of a plasmid expressing an anti-VEGF receptor to endothelial cells and the RPE of choroidal neovascular lesions in rats [72]. PLGA NPs loaded with brinzolamide effectively reduced IOP in rabbits, releasing the anti-glaucomatous drug up to 10 days after their subconjunctival injection [73]. Overall, the use of PLGA as a drug-vehicle presents some drawbacks, including weak protein stability, suboptimal loading efficiency, and abrupt drug release.

Bourges et al. tested the intravitreal injection of PLA NPs loaded with fluorochromes in rats. They observed a sustained presence of fluorochromes in the mice’s retinal cells for 1 month, with detectable levels in the ganglion cells for up to 4 months [74]. Dexamethasone loaded on PLA NPs and administered intravenously to uveoretinitis model rats was demonstrated to be effective in controlling inflammation [75].

PCL, a polyester derived from polymerization of caprolactone, can also be loaded with dexamethasone. This implant provided a therapeutic concentration of dexamethasone for one year in rabbit eyes [76]. Immunosuppression with cyclosporine loaded in PCL NPs was studied by Yenice et al. They reported a drug bioavailability 10 to 15-fold higher with this formulation than with its solution in castor oil [77].

PCL copolymerization with PEG, forming PEG-PCL-PEG polymers, resulted in a biocompatible triblock nanocarrier that was potentially effective in retinal drug delivery, whereas combinations of PCL with PLA or PGA were seen to induce permeability and crystallinity alterations [78].

The first NPs for ocular drug delivery were polymers of PBCA, following their broad use as drug delivery carriers [79]. PBCA NPs present favorable characteristics for this function, including stability, biocompatibility, biodegradability, targetability, and the capability to cross the blood–brain barrier when coated with polysorbate 80 [80,81,82,83,84,85]. In vivo confocal neuroimaging (ICON) allowed some authors to be the first to record in real time the NP crossing of BRB [86,87,88]. The effects of the nanoparticles’ size and zeta potential on their capability to trespass the biological barriers were also studied, showing that larger PBCA NPs (272 versus 172 nm) with intermediate z potential (5 V versus 0 V and 15 V) were more likely to concentrate in the retina. Their accumulation in RGCs, together with their well-documented safety profile in humans, makes this polymer a viable option for delivering retinal therapies [89,90].

#### 4.1.6. Chitosan and Chitosan-Based Nanotechnologies

Chitosan (CS), a highly diffused polysaccharide (the second most abundant in nature), represents an important structural component of the exoskeleton of several insects, crustaceans, and fungi. This substance has been used as a penetration enhancer in pharmaceutical research and industry for decades [91]. The favorable properties of CS include the absence of cytotoxicity, high biocompatibility and biodegradability, and a polycationic and mucoadhesive nature. For these reasons, the addition of CS to ocular topical drugs represents a safe and effective strategy for prolonging the drug presence in the precorneal area and making the active drug more bioavailable. Several studies have been conducted on the use of CS and its derivatives for drug-loaded NPs, liposomes, and solid lipid matrixes to improve their adhesion and penetration across ocular tissues.

##### Chitosan Nanoparticles

The encapsulation of ophthalmic drugs in CS-coated NPs and their administration in solution have been shown to prolong their residence time in the precorneal area [92,93,94]. NPs for ocular formulations generally do not exceed 10 µm to avoid foreign body sensation and ocular irritation. CS NPs are generally spherical and smooth, therefore possessing an optimal surface area-to-volume ratio that also enhances their reactive surface and therapeutic potential. Several methods have been proposed to prepare different types of CS NPs, such as ionotropic gelation, spray drying, water-in-oil emulsion crosslinking, reverse micelle formation, emulsion droplet coalescence, nanoprecipitation, or self-assembly, with the first usually preferred due to its relative simplicity and convenience. Several drugs have been loaded in CS NPs to treat different diseases in the posterior segment of the eye, including implants containing bevacizumab-loaded CS NPs [95] and PLGA microparticles entrapping ranibizumab-loaded C NPs [96] for the treatment of choroidal neovascularization (vide infra), CS NPs for delivery of daptomycin in bacterial endophthalmitis, although showing lower antimicrobial susceptibility as compared to the antibiotic in its free form [97].

Carboxymethyl chitosan (CMCS) was used by Wang et al. to coat nanocomposite carriers used to deliver dexamethasone (DEX) to the posterior pole. Topical eyedrops containing coated nanocomposites, non-coated nanocomposites, and commercially available DEX were instilled in albino rabbits. Retina-choroid homogenates were then analyzed using high-performance liquid chromatography (HPLC), showing higher concentration and longer permanence of DEX when delivered by CMCS-coated nanocomposites than by non-coated composites or commercial DEX eyedrops. The nanocomposite absorption was seen to occur mainly via the conjunctival-scleral pathway using in vivo fluorescence imaging. The cellular uptake was studied in vitro on human conjunctival cells, and appeared to be energy dependent, consisting of clathrin-mediated endocytosis, as demonstrated by a 54% reduction in cellular uptake in the presence of specific inhibitors of this clathrin-mediated mechanism [98].

Polymeric nanocarriers made of CS-grafted poly(ethylene glycol) methacrylate (CS-g-PEGMA) were loaded with bevacizumab in a study by Savin et al. The authors crosslinked the polymer using either sodium tripolyphosphate (TPP) or Na_2_SO_4_, forming 200–900 nm or 1000–1500 nm NPs due to lower and higher molecular crosslinking density, respectively, and obtained a prolonged and sustained drug release with a small burst effect [99].

##### Chitosan Micelles

CS has been tested as a component of polymeric micelles to facilitate the ocular release of several ocular drugs. Xu et al. synthesized a CS derivative (CS oligosaccharide-valyvaline-stearic acid) to prepare dexamethasone-loaded nanomicelles (30 nm) for the topical treatment of macula edema [100].

The presence of the oligosaccharide was a key factor in reaching the posterior ocular pole via the conjunctival route, and the positive electric potential on the naonomicelle surface (+30 mV) favored their adhesion to the anionic ocular surface. In vitro analysis demonstrated sustained drug release without bursts and no toxic effects.

##### Chitosan Lipid Nanoparticles and Liposomes

As a polysaccharide, CS can be used to prepare lipid NPs (LNPs) and liposomes. LNPs consist of a lipidic core stabilized by a surfactant on the surface that stabilizes the particle in an aqueous environment. These characteristics make LNPs potential vehicles for lipophilic molecules by all administration routes, although their drug-loading efficacy is relatively poor. As for micelles, their content can be expulsed rather easily during storage.

Different from LNPs, liposomes consist of a double surfactant layer, structurally resembling a living cell, that can include both lipophilic and hydrophilic compounds and makes these particles highly biocompatible and biodegradable with low inner toxicity. Li et al. tested the ocular penetration enhancement effects of a CS coating on liposomes containing triamcinolone acetonide (TA), an intermediate-acting corticosteroid employed to treat ocular inflammatory and angiogenic conditions. After topical administration to mice, the presence of TA in the posterior segment was tested using optical coherence tomography (OCT). The OCT scans showed a stronger signal from the retinal surface of mice treated with CS-coated liposomes than in controls receiving non-coated liposomes, although the difference in signal strength was not significant. The TA effect on the signal was detectable 10 min after instillation, peaked at 6 h, and lasted up to 12 and 10 h in subjects receiving coated and non-coated preparation, respectively, thus indicative of a chitosan effect on the TA permanence time [101]. The effectiveness of these CS-coated liposomes was further studied by Cheng et al., showing their efficiency in relieving laser-induced retinal edema without toxic effects [102].

Similar results were observed by Khalil et al., who prepared CS-coated liposomes encapsulating TA, and administered as topical treatment to rat models. After 15 days, the drug was detectable in the ocular posterior chamber in vivo [103].

##### Chitosan in Combined Drug Delivery Systems

The drug targeting and release modulation of CS-containing nanocarriers can be further ameliorated by combining them with other delivery vehicles, such as polymeric coatings, larger molecules, gels, lenses, and inserts. PLGA has been experimentally associated with CS for retinal drug delivery. Elsaid and his collaborators formulated a system-within-system matrix consisting of PLGA microparticles containing CS-based nanoparticles to deliver ranibizumab to the vitreous. The authors showed that the presence of CS enhanced ranibizumab loading and release thanks to the bonds between the drug and the nanoparticles [96].

NPs loaded with the anti-VEGF molecule bevacizumab were inserted by Badiee et al. into a hyaluronic acid (HA)/zinc implant. The system presented a homogeneous NP distribution and offered sustained drug release over two months in vitro [95].

A CS-based injectable hydrogel for the delivery of retinal progenitor stem cells was fabricated by Jiang et al. for the treatment of retinal degeneration. Oxidized dextran (Odex) was bonded with the CS amine groups to form the hydrogel. The proliferation of retinal progenitor cells and their differentiation toward neurons was favored by the CS/Odex hydrogel [104].

A modified CS-based hydrogel was synthesized by Moreno et al. for the administration of ranibizumab and adflibercept, two for neovascular AMD and corneal neovessels. The investigators created a thiolated, more densely cross-linked hydrogel capable of retaining the drug-load molecules for a more prolonged time, preventing burst release in vitro [105].

Abe et al. created a silicone-made refillable reservoir for transscleral drug delivery containing 1% CS and 3% gelatin matrix for the treatment of retinal diseases. The gel itself resulted in a 5-day drug release, with an initial burst, whereas its insertion in the reservoir extended it by 2–5 times. In vitro, and up to 12 weeks in vivo [106].

#### 4.1.7. Metallic and Other Inorganic Nanomaterials

Gold, silver, and platinum NPs have shown important therapeutic properties. The ability to permeate the BRP seems to be size dependent. Gold NPs of a 20 nm size can cross this barrier, even after intravenous administration, differently from 100 nm NPs [107]. Silver NPs demonstrated anti-angiogenic effects in AMD models. Vascular permeability is also impaired by these nanoparticles, interfering with pathologic endothelial mechanisms occurring in retinal diseases, such as retinopathy of prematurity (ROP), RP, and DR [108,109]. Similarly, gold NPs were seen to contrast angiogenesis and inflammation in retinal pathologies [110]. Intravitreal injections of magnetic nanoparticles (MNPs) delivered neuronal growth factors (NGF) to the retina for neuroprotection in Xenopus embryos. In detail, injecting MNPS-GNF complexes prevented retinal ganglion cell apoptosis, whereas injection of free GNF did not [111].

Cerium NPs, also called nanoceria, delivered by intravitreal injection, protected retinal cells from oxidation, caspase-induced apoptosis, and retinal degeneration in AMD model mice [112,113]. A decrease in rod cell apoptosis and an augment of retinal lipid peroxidation were obtained in RP model rats treated with nanoceria, proving their important antioxidant properties and potential application in other types of retinal degeneration [114,115]. Nanoceria were detectable for more than 1 year after its intravitreal injection in murine models, without development of inflammatory or other pathological side effects, indicating a sustained and safe action of this type of NP [116].

Silicate antiangiogenic properties emerged from studies on intravitreal silicate-based NPs in mice with oxygen-induced retinopathy [117]. A helical vector containing a silica scaffold and a magnetic element of iron or nickel was experimented with to deliver therapeutic substances to the retina. The vector was precisely moved through the vitreous applying a magnetic field ab externo coated in perfluorocarbon liquid to reduce attrition, and its position was monitored in real time by OCT imaging [118].

Relevant potential risks in using inorganic materials in vivo derive from their poor or absent biodegradation or clearance [119]. Metal nanoparticles 20 to 80 nm in diameter showed toxic effects on photoreceptors in vitro and damage to the blood–retinal barrier derived from intravitreal injection of NPs containing titanium [120,121]. Therefore, further studies are required to assess and optimize the safety of therapeutic delivery systems based on these materials.

#### 4.1.8. Encapsulated Cell Technology

A new technology using encapsulated human RPE cells has been developed by Neurothec Pharmaceuticals. NT-501 (Renexus^®^) consists of an implantable polymeric scaffold, encapsulating cells that are genetically modified to secrete ciliary neurotrophic factor (CNTF). This capsule-containing cells is composed of a semipermeable membrane surrounding a scaffold made of polyethylene terephthalate strands, which protects the cells from the immune system while allowing the passage of nutrients and therapeutic molecules. A titanium loop is attached to the extremity of this capsule, allowing anchorage to the scleral wall after surgical implantation via the pars plana. Initially developed to treat RP and dry AMD, this device has also been considered in the management of patients with glaucoma and type 2 macular teleangectasia. Studies have reported that NT-501 slowed retinal degeneration and stabilized patients’ reading speed [122,123,124].

### 4.2. Other Topical Absorption Enhancers

Technologies to enhance the absorption of topical drugs have recently emerged as a promising solution to increase bioavailability. Ocular penetration enhancers (PEs) are chemical or biological agents that are associated with topical drugs, such as excipients or additives, and are used to increase the ability of the active drug to overcome ocular barriers and penetrate the eye. Several types of PEs have been reported in the literature, and most of them limit the drug delivery action to the anterior chamber. Benzalkonium chloride (BAC) and different types of cell-penetrating peptides, however, have shown significant results in internalizing drugs across cellular membranes to the posterior segment [125,126,127,128]. These peptides have been initially studied to carry drugs across skin, respiratory tract mucosae, blood–brain barrier (BBB), and, more recently, into the ocular tissues.

#### 4.2.1. Cell-Penetrating Peptides (CPPs)

CPPs are short-chain peptides, usually composed of 30 or less amino acid residues, capable of trespassing membranes with no need for chiral interactions with surface receptors. The transactivator of transcription (TAT), a protein transduction domain (PTD) and one of the most studied CPPs, was investigated by Wang et al. to deliver growth factors to retinal tissue in vivo. The group performed studies in which TAT was conjugated with acidic fibroblast growth factor (aFGF) and topically administered by eyedrops to rats with retinal ischemia reperfusion. Immunohistochemical (IHC) studies documented the presence of TAT-aFGF in retinal tissue 30 min after instillation. The major concentration was observed in the ganglion cell layer (GCL), suggesting a cell-specific uptake mechanism of TAT, with a peak between 30 and 60 min after administration and detectable presence for up to 8 h. The histological and electrophysiological analysis of the treated eyes also identified a reduction in GCL apoptosis and a faster functional recovery compared to non-treated and aFGF-only treated eyes. The absence of TAT-aFGF in the deeper corneal stroma and endothelium indicated to the authors the involvement of a non-corneal absorption route [129].

Zhang et al. also reported that TAT is an effective carrier for treating retinal diseases in animal models. They conjugated TAT to endostatin (Es), an inhibitor of choroidal neovascularization (CNV), and topically administered TAT-Es to experimental mice. IHC exams revealed a good retinal distribution of TAT-Es in treated subjects, and a significant reduction in their CNV areas in comparison to controls and to mice treated with unconjugated Es. Furthermore, the CNV area reduction obtained with topical TAT-Es was comparable to that induced in controls receiving intravitreal bevacizumab and intravitreal TAT-Es. Endocytosis, majorly micropinocytosis, was suggested as the principal uptake mechanism in this study, with clathrin- and caveolae-mediated endocytosis and energy-independent direct translocation serving as secondary mechanisms [130].

TAT conjugated with Es or with Es and an arginine-glycine-aspartic tripeptide (RGD) was tested by Li et al. on experimental mice with oxygen-induced retinopathy. The investigators aimed at verifying the inhibitory properties of this fusion protein on angiogenesis and neo-vessels formation. The retinal concentration at 1 h from topical instillation was seen to be 14-fold higher than Es alone by ELISA testing, with TAT-Es-RGD slightly more concentrated than TAT-Es, due to the specific RGD binding properties with the integrins on the angiogenic endothelial cell surface. Histologically, the investigators observed the significant angio-inhibitory effects of topical TAT-Es and TAT-Es-RGD, as a reduction of vessel tufts and avascular areas on fluorescein microscopy, and as a reduction in endothelial cell nuclei breaking through the inner limiting membrane on microscopy. No difference was observed between the groups of mice treated with non-conjugated Es and Es-RGD and the control group of untreated mice. Moreover, vascular endothelial growth factor (VEGF) levels detected by IHC similarly dropped in response to topical TAT-Es-RGD and intravitreal anti-VEGF [131].

In a study by Chu et al., dual TAT-RGD peptides were bound to PEG-PLGA nanoparticles (NP) to enhance the penetration in murine eyes affected by laser-induced CNV. Confocal microscopy of choroid-and-retina samples from the CNV areas revealed the highest NP presence, expressed as fluorescence intensity, in mice treated with eyedrops containing TAT-RGD-NPs, when compared to mice receiving topical NP alone (11-fold higher), with TAT-NP and RGD-NP preparations resulting in intermediate fluorescence intensities. RGD was confirmed in this study to concentrate specifically in the CNV area due to its affinity with the endothelial integrin α-V β-3 [132].

Retinal protection was obtained in rats by Atlasz et al. by topical administration of TAT bound to two peptides: vasoactive intestinal peptide (VIP) and pituitary adenylate cyclase-activating polypeptide (PACAP), with considerable retinoprotective and anti-inflammatory activity, respectively. Similar to other studies, conjugation with TAT allowed these therapeutic molecules to target retinal cells more efficiently. This was demonstrated by fluorescence microscopy, which showed a much higher fluorescence per retinal unit area in rats receiving PACAP-TAT/ VIP-TAT eyedrops in comparison with rats receiving PACAP/VIP. The authors also calculated the efficiency for traversing the eye to the retina, finding it 3-fold higher in PACAP-TAT/ VIP-TAT than in PACAP/VIP [133].

Penetratin, another CPP derived from *Drosophila melanogaster*, has been reported to act as a drug PE in several studies. Liu et al. were the first to report the penetration efficiency and biodistribution of topically administered penetratin in both anterior and posterior ocular structures. Fluorescence microscopy showed internalization of this agent within 10 min of the eyedrop instillation, with maximum uptake in the photoreceptors and RPE layers. Accumulation was detectable for up to 6 h, with a peak around 30 min after administration. Penetratin also showed low conjunctival cytotoxicity in vitro, with an IC50 value of 2.5 mM, which was lower than TAT IC50 (2 mM), and 100% of cellular viability even at concentrations as high as 30 mM. Penetration into the posterior segments tends to be favored by its cationic and amphipathic nature, in addition to the helicoidal structure (polyproline type 2) of this agent [134].

Further studies by Liu et al. demonstrated the penetration and retinal localization of a topical preparation of penetratin conjugated with polyamidoamine (PAMAM) dendrimers delivering red fluorescent protein plasmid (pRFP) in retinal cells. Fluorescence microscopy detected penetratin-PAMAM-pRFP in the posterior segment after 10 min, with an accumulation peak at 1 to 2 h and persistence for up to 8 h. The molecular complex was mostly observed in photoreceptors and the RPE, in which the fluorescence was more intense. Similarly, the transfected gene expression was evident in photoreceptors, inner plexiform, and outer plexiform layers and absent in control subjects treated with non-conjugated plasmid [135].

Penetratin hydrophobic derivates were seen to perform even better by Jiang et al., who compared them with wild-type penetratin and penetratin hydrophilic derivates. All three types of peptides, administered in vivo by eyedrops, showed penetration in the anterior and posterior ocular segments after 10 min, peaking at 1 h. Hydrophobic derivates reached the highest retinal concentration among them, resulting in the most intense fluorescence on microscopy. Hydrophilic derivates, however, resulted in a weaker fluorescence compared to the wild-type penetratin. As a possible explanation for these findings, the authors suggested a higher presence of a helix structure in the hydrophobic peptides that could facilitate the interaction with biological membranes and permeation across them. In vitro toxicity assays on human corneal and conjunctival cells, using MTT, and ex vivo biocompatibility testing on excised rabbit corneas and sclera also showed good results [136].

Yang et al. conjugated penetratin and RGD peptide with PEG-PAMAM dendrimers nanocarriers (NCs) and demonstrated that these 2 peptides enhanced ocular permeation and posterior segment localization after topical ocular administration in mice. Conjugation of NCs with penetratin or RGD-penetratin resulted in higher peptide retinal concentration, evident as a 3 times more intense retinal fluorescence, and a prolonged permanence in the retinal tissue, expressed as a longer fluorescence emission (24 h vs. 12 h), in comparison to mice receiving non-conjugated NCs. No cytotoxicity emerged in vitro by testing these NCs on human corneal epithelial cells and umbilical vein endothelial cells. PEG conjugation seemed to reduce PAMAM cytotoxicity, preventing direct contact between cells and amines [137].

A specific peptide for ocular delivery (POD) was investigated by Johnson et al. to vehicle drugs to the posterior segment in vivo. They instilled this carrier-drug complex in mice as topical eyedrops, and by using microscopical studies, the authors revealed localization of this agent in the sclera, choroid, and the optic nerve dura after 45 min, being eliminated at 24 h. No localization was observed in the controls treated with the non-conjugated drug. The uptake seemed to be temperature dependent, whereas inhibitors of endocytosis in vitro did not stop internalization, excluding endocytosis as a key element [138].

A novel CPP based on poly-arginine was tested by de Cogan et al. to deliver anti-VEGF in animal models. Topical treatment was administered to Sprague-Dawley rats and wild-type mice populations to study the pharmacokinetic and angio-inhibitory aspects of the CPP-drug complex, respectively. ELISA testing revealed a 10–11 times higher drug concentration in the target tissue when the anti-VEGF was conjugated with CPP. The angio-inhibitory effect CPP drug was recorded as a reduction of the CNV and scarring area using fluorescein angiography (FA), IHC, and infrared imaging on choroid/RPE samples. A similar response was achieved with intravitreal anti-VEGF, whereas non-treated mice and those receiving the non-conjugated anti-VEGF eyedrops showed no response. No cytotoxicity emerged when this compound was tested on murine retinal cells, human ARPE-19 cells, and human corneal fibroblasts in vitro [139].

#### 4.2.2. Cyclodextrins

Cyclodextrins (CDs) are a group of oligosaccharides composed of six (α-CD), seven (β-CD) or eight (γ-CD) glucose molecules. They possess a truncated cone shape, with a more lipophilic central cavity and a more hydrophilic outer surface. This configuration provides protection from degradation and potential environmental detrimental factors (e.g., reactive molecules). The solubility in water of CDs in their natural forms is relatively poor but can be artificially enhanced to derive molecules such as 2-hydroxyoropyl-β-CD (HPβCD), sulphobutyl ether-CD (SBEβCD), methylated-β CD (MβCD) and 2-hydroxypropyl- γCD (HPγCD), which can be used to augment the topical absorption of hydrophobic drugs. The concentration of dexamethasone in the anterior and posterior ocular segment has been studied by Sigurdsson et al. The study reported that two hours after topical ocular administration of 0.5% dexamethasone/MβCD aggregates in albino rabbits, the drug retinal concentration were 33  ±  7 ng/g in the study eye vs. 14  ±  3 ng/g in the control eye, indicating that 19 ng/g (58%) was derived from topical absorption and 14 ng/g (42%) from systemic absorption. In the vitreous and optic nerves, the portions of dexamethasone topically absorbed were 55% and 17%, respectively [140]. Loftsson et al. compared the ocular absorption in albino rabbits of 1.5% dexamethasone either aggregated with MβCD (solution) or γCD (suspension). The dexamethasone 1.5%/γCD aggregated suspension resulted in 49% of retinal dexamethasone derived from topical administration vs. 14% using the dexamethasone 1.5%/MβCD aggregates solution, whereas in the vitreous humor, the portions of drug coming from the topical instillation were 86% and 73%, respectively, for these two preparations. In the study, the authors observed that suspensions containing dexamethasone/γCD aggregates effectively targeted the ocular posterior segment, especially the retina, and that γCD limits the systemic absorption of the drug by 80% [141].

Clinical studies involving CDs in the topical treatment of diabetic macula edema have been conducted by Tanito et al. The investigators administered 1.5% dexamethasone/γCD eyedrops 3 times per day in 19 patients affected by diabetic macular edema, obtaining a significant central macular thickness (CMT) reduction at week 4 from 512  ±  164 µm to 399  ±  154 µm (*p*  =  0.0016), with a ≥ 10% reduction in 12 out of 19 patients and a mean reduction of −20.3% (range −65.4% to  +  9.8%), significantly higher in vitrectomized (−47%) than in un-vitrectomized (−15%) eyes (*p*  =  0.009). LogMAR VA also significantly improved from baseline (mean  ±  SD) 0.52  ±  0.41) to week 4 (0.37  ±  0.40) (*p*  =  0.0025) with 14 of 19 eyes (74%) improving by more than 0.1. Intraocular pressure slightly increased from (mean  ±  SD) 15.2  ±  3.1 mmHg at baseline to 17.4  ±  4.2 mmHg (*p*  =  0.0015) at week 4, then returning to pretreatment values at week 8 (15.8  ±  4.0) [142].

In a randomized controlled trial, Ohira et al. compared the effects of topical 1.5% dexamethasone/γCD (12 eyes) tapered every 4 weeks from 3 to 2 and finally to 1 time per day, and, as a control, the results of one subtenon injection of 20 mg of triamcinolone acetonide (10 eyes) in patients with diabetic macular edema. Both LogMAR VA and CMT significantly improved in both groups from baseline to 4 weeks (treatment group: from Log MAR 0.41 ± 0.3 to 0.09 ± 0.15, *p* < 0.05; from 483 ± 141 µm to 99 ± 169 µm, *p* < 0.05; control group: from Log MAR 0.42 ± 0.28 to 0.1 ± 0.14, *p* < 0.01; from 494 ± 94 µm to 106 ± 88 µm, *p* < 0.001) and to 8 weeks (treatment group: Log MAR 0.11 ± 0.13, *p* < 0.05; 141 ± 211 µm, *p* < 0.01; control group: Log MAR 0.08 ± 0.16, not significant; 106 ± 92 µm, *p* < 0.001). CMT was reduced  ≥ 20% in one-half of the topically treated patients and 60% of the controls. VA improved by 2 or more lines in 42% of the topically treated patients vs. 20% of the control group. A modest IOP elevation was detected in the treatment group only at 4, 8, and 12 weeks, whereas both groups showed a transient lowering in cortisol and adrenocorticotropic hormone (ACTH), indicating systemic absorption, and lasting, in the topical therapy group, up to 4 weeks after the treatment suspension. No significant glycemic alterations were observed. This study confirmed the anatomical and functional improvements of topical 1.5% dexamethasone/γCD aggregated suspensions, which were comparable to those obtained with a sub-tenon injection of triamcinolone acetonide [143].

Studies have reported noninfectious posterior uveitis treated with 1.5% dexamethasone/γCD aggregated suspension. Shulman et al. administered this preparation in 5 uveitic patients (1 female and 4 males, age range 45–65 years) affected by vitritis (3 eyes) and macular edema (5 eyes), starting with 4 instillations per day and tapering the drug by 1 drop per day every 10 days. The study was open-label and non-comparative. Macular edema completely resolved in 4 eyes, relapsed at week 12 after initial response in one case. VA improved in 3 eyes, remained stable in 3 eyes, and decreased in the other 2 eyes, also because of cataract in one of them, with median VA significantly improved at week 4, but returning to baseline values at 12 weeks. Vitritis completely resolved in 2 eyes, but improved in the third. One patient, who was known to be a steroid-responder prior to the study, actually presented with IOP elevation after the treatment [144].

Uveitis and macular edema were also treated with eyedrops containing 1.5% dexamethasone/γCD particles in studies by Krag & Hassellund. They administered the topical preparation in 3 cases. The posology was 4 times daily for one month in the affected eye in one case, obtaining macular edema reabsorption, therefore switched to conventional topical dexamethasone, with edema recurrence after one month. The second case was bilateral: one eye was treated with 2 drops of preparation, instilled 4 times per day for three weeks, then tapered to 1 drop 3 times per day, whereas the other eye underwent an intravitreal implant of Ozurdex. Both eyes responded well, showing edema resolution. The third patient also presented with bilateral uveitic macular edema, which had recurred in both eyes 3 months after intravitreal Ozurdex. The recurrence was treated in one eye with topical 1.5% dexamethasone/γCD 6 times daily for one week, then 4 times daily for another week, but without success. The CME augment required a second Ozurdex implant [145].

#### 4.2.3. BAC

BAC is a widely used preservative in ocular topical formulations. It acts as a PE by damaging the anatomical barriers of the ocular surface and facilitating the penetration of solutes and solvents through the external ocular epithelia. Studies on nanoparticles administered via eyedrops in association with BAC were performed on mice by Mahaling and Katti. The enhancement power of BAC was not as strong as expected on fluorescence biomicroscopy, although increased bioavailability of BAC-coated nanoparticles was recorded in the retina, choroid, and sclera compared with non-coated nanoparticles [146].

#### 4.2.4. Iontophoresis

Iontophoresis can also be used to transport drugs across ocular membranes with a non-invasive mechanism. This technique uses a low-amplitude electrical current delivered in a continuous or pulsatile way without damaging the ocular tissues. Iontophoretic treatment for the posterior segment forces the passage of dedicated topical drug formulations through the transscleral pathway to avoid anterior anatomical barriers.

Several preclinical studies have shown interesting results in delivering corticosteroids to the inner ocular structures, including the vitreous, choroid, and retina. Hemisuccinate methylprednisolone (HMP) was administered to rabbits using transscleral iontophoresis, reaching higher and more sustained concentrations than with intravenous administration, and with minimal systemic absorption and ocular complications [147].

Cathodal transscleral iontophoresis, experimented on rabbits, allowed us to obtain intraocular concentrations of topical DEX higher than those obtained via passive diffusion of the same preparation. The concentration measured in the vitreous was well above the concentration needed to suppress inflammation [148].

Preliminary encouraging results on the transscleral iontophoretic administration of dexamethasone phosphate and its favorable molecular characteristics (high water solubility, 2 negative charges at physiologic Ph) led to the development of dedicated patented devices, such as the EyeGate II delivery system (EGDS; EyeGate Pharmaceuticals, Inc, Encintas, CA, USA.). The device contains an electrode housed in an annular ocular applicator, which produces ions that push the drug, a dedicated dexamethasone phosphate formulation, through the conjunctiva and sclera by electrochemical repulsion [149].

A phase-1 clinical trial has recently started to deliver bevacizumab and ranibizumab using an iontophoretic device called Visulex-1; however, concerns about skin exposure to prolonged electric currents have been reported [150,151,152].

### 4.3. Sustained Drug-Release Systems

#### 4.3.1. Ocular Inserts

Ocular inserts (Ois) are sterile, non-implantable, thin, multilayered routes of administration with a solid or semisolid consistency developed to achieve better ocular bioavailability and sustained drug action over a prolonged period [153,154]. They have been designed to overcome eyedrop limitations and increase the contact time between the preparation and the conjunctival tissue to ensure a sustained and accurate release suited for topical or systemic treatments [155]. They are placed in the fornix of the conjunctival sac of the lower eyelid and, less frequently, in the upper fornix or on the cornea, offering an alternative approach to the difficult problem of limited pre-corneal drug residence time [156]. Such systems can achieve prolonged therapeutic drug concentrations in ocular target tissues while limiting systemic exposure and side effects and improving patient adherence to therapy [157]. Based upon solubility, they can be classified as insoluble (osmotic, diffusion, contact lens); soluble (based on natural polymers, e.g., collagen, or based on synthetic or semi-synthetic polymers, e.g., cellulose derivatives, such as HPMC, HPC, MC, etc.); and bioerodible [158].

Ois have been used to treat conditions, such as glaucoma, Ocusert ® (pilocarpine-alginate), NODS ®, New Ophthalmic Delivery System (pilocarpine) dry eye, and allergy [159,160,161,162]. In addition, Ois has been utilized for the treatment of common ocular infections, such as bacterial keratitis [163,164,165]. Ois loaded with cyclosporine (Cys) has been reported by Grimaudo et al. [166]. Ois based on sodium hyaluronate (HA) nanofibers loaded with the antioxidant compound ferulic acid (FA) and the antimicrobial peptide ε-polylysine (ε-PL) have shown high antibacterial activity against Pseudomonas aeruginosa and Staphylococcus aureus [163,164,165,166,167]. Other Ois have been developed and loaded with Ketorolac Tromethamine (KT) Eudragit ®, Atorvastatin Cakcium (ATC) to treat ocular inflammatory conditions [168,169,170,171]. Moreover, Ois offers the possibility of targeting internal ocular tissues through non-corneal conjunctival-scleral penetration routes to reach the ocular posterior segment. A new technique used to develop new synthetic Ois is electrospinning, which is a method that obtains nanofibers that have the property to encapsulate and control the release profile of multiple drugs [172]. Nanofibers, due to their unique structural features, show great promise for drug delivery to the retinal segment following topical application. Preclinical results have shown that preservative-free polycaprolactone (PCL) electrospun nanofibers can be considered a primary drug carrier for the delivery of fluocinolone acetonide into the posterior eye segment, particularly for retinal epithelium [173]. The melt-cast method has been used to fabricate topical Ois for the delivery of indomethacin, prednisolone sodium phosphate, and ciprofloxacin hydrochloride commonly employed in the treatment of ocular inflammation and infections. Studies have shown that these devices generated significantly higher drug levels in all ocular tissues, including the retina-choroid, when compared to their control formulations, demonstrating that the melt-cast/melt-extruded films could shift the paradigm for drug delivery to the posterior segment of the eye [174]. More recently, the effects of Ois for the administration of progesterone (PG), a sexual hormone characterized by neuroprotection activity and used for the treatment of posterior degenerative ocular diseases associated with oxidative stress, have been evaluated using permeation enhancer technology. Due to the fact that the PG is a hydrophobic drug, it was incorporated in β-cyclodextrins in order to solubilize it. A controlled diffusion of PG was obtained by in vitro grafting of 59% polyvinyl alcohol (PVA), 39% polyvinylpyrrolidone K30 (PVP-K30), and 2% propylene glycol (PGL). Ex vivo analyses assessed trans-corneal and trans-scleral diffusion of PG and allowed the authors to conclude that the PG-loaded Ois can be suitable for the treatment of various posterior eye diseases such as DR, AMD, cataracts, glaucoma, and retinitis [175]. Ois are placed in the conjunctival fornix or on the cornea and increase the contact time between the drug and the conjunctival/corneal tissue in order to ensure a long-lasting, controlled, and precise release of the preparation. In this way, they overcome the problem of limited pre-corneal drug residence time, leading to increased bioavailability. By avoiding the use of preservatives, they decrease the likelihood of sensitivity reactions and minimize systemic side effects. Other advantages include precision dosing with controlled release and minimal systemic absorption, reduced administration frequency, and improved patient compliance. Electrospinning allows nanofibers to be obtained that encapsulate and control the release profile of numerous drugs to the posterior eye segment [172]. Moreover, the melt-cast method allows the generation of higher drug levels in ocular tissues, including the retina and choroid [174]. Despite these advantages, the major disadvantages of Ois reside in patient non-compliance with frequent feelings, such as the entry of a foreign body into the eye, difficulty in self-insertion or removal, potential accidental loss and movement around the eye that could interfere with vision. Ois can be specifically formulated to address the unique characteristics of retinal diseases, such as the need for long-term treatment and the delicate nature of the retina. Research and development in this area is ongoing, with a focus on optimizing the design, drug release, and biocompatibility of ocular inserts for the treatment of retinal diseases. These advancements hold great promise in improving the outcomes and quality of life of patients with retinal conditions.

#### 4.3.2. Ocular Implants

Intraocular/intravitreal implants are relatively new routes of administration designed to be inserted into the eye to achieve a sustained release mechanism of the drug in the vitreous and to provide a long-term therapeutic effect in a controlled and prolonged way. They can bypass the BRB, avoid burst release, provide a reduction in the dose, and deliver therapeutics at a constant rate directly at the ocular site, with no need to perform repeated injections intravitreally. These implants have an increased half-life, reduced peak plasma levels, and improved patient compliance [176,177]. The mechanism of drug release from implants showed three phases, which include an early burst, a middle diffusive phase, and a final burst. There are two major categories of materials used in the development of these implants: non-biodegradables and biodegradables.

Non-biodegradable implants (NBI), such as scleral, intra-scleral (disc), and intravitreal implants (encapsulated cell), do not experience changes in structure and require two surgical procedures, which entails one for implantation and a second one for removal or replacement. They are larger in size, thus requiring a larger incision for implantation. They are made up of ethylene vinyl acetate (EVA), polyvinyl alcohol (PVA), or polysulfone capillary fiber (PCF).

Biodegradables implants (BI), such as injectable microparticles, intravitreal implant (injectable rod), intra-scleral, and epi-scleral implant (disc, scleral implant (plug), degrade and disintegrate over time. All the components from the implant are eliminated autonomously; thus, only a single surgical procedure is needed for insertion [176]. They are made of polylactic acid (PLA), polyglycolic acid (PGA), PLGA, or polycaprolactones.

With regards to the currently available NBIs, the first insert was developed and approved in 1996 Vitrasert^®^ (Bausch and Lomb, Rochester, NY, USA) was designed for the treatment of cytomegalovirus retinitis with ganciclovir. It has been demonstrated to control inflammation, reduce recurrences in patients with viral retinitis, and improve visual acuity [40,178]. Retisert (Bausch & Lomb, Rochester, NY, USA) is an intravitreal NBI of a corticosteroid, fluocinolone acetonide, which the FDA approved in 2005 for treating chronic non-infectious uveitis [179]. The main indications for this insert include chronic non-infectious posterior uveitis (NIPU), but it has also been shown to be effective in DME and macular edema (ME) secondary to central retinal vein occlusion (RVO) [180,181]. Iluvien (Alimera Sciences Inc., Alpharetta, GA, USA) is the smallest NBI of FA. It is cylindrically shaped and has a composition similar to Retisert. This insert is used to treat DME but is also currently being evaluated in phase 2 trials for dry AMD, retinal vein occlusion, and non-infectious uveitis [182,183].

Yutiq (EyePoint Pharmaceuticals, Inc., MA, USA) is similar to Iluvien and is another intravitreal NBI of FA that received FDA approval in 2018 for the treatment of chronic NIPU [184]. I-vation (SurModics, Eden Prairie, MN, USA) is an NBI that releases triamcinolone acetonide (TA) and is used to treat DME [185,186]. The Ranibizumab Port Delivery System (PDS) is a new intraocular DDS for continuously delivering an anti-vascular endothelial growth factor (VEGF) antibody, ranibizumab, for the treatment of neovascular AMD (nAMD). It is made up of an ocular implant and four ancillary devices that are utilized for initially filling ranibizumab, surgical implantation, refilling exchange, and explantation [187].

The DEX implant (Ozurdex, Allergan Inc., Irvine, CA, USA) is a dexamethasone intravitreal implant composed of poly lactic-co-glycolic acid (PLGA), a synthetic aliphatic polyester predominantly biodegraded via non-enzymatic hydrolysis of the ester linkages under physiological conditions. It contains 0.7 mg dexamethasone, which is released sustainably in the vitreous for up to 6 months. This device received FDA approval in June 2009 for treating retinal vein occlusion-associated macular edema and in 2010 for treating non-infectious posterior uveitis. The main indications include RVO (central and branch) associated with ME, DME, and NIPU. It also has off-label use in Irvine Gass syndrome, nAMD, vasoproliferative retinal tumors, retinal telangiectasia, Coats’ disease, radiation maculopathy, retinitis pigmentosa, and macular edema secondary to scleral buckle and pars plana vitrectomy [188]. The DEX implant offers prolonged and targeted drug delivery and is placed directly into the vitreous to maximize its therapeutic effect while minimizing systemic side effects. By delivering a consistent and controlled amount of dexamethasone, it can maintain therapeutic drug levels over an extended period. Moreover, patients may experience a reduced treatment burden compared to other forms of medication, thus improving their convenience and compliance. On the other half, potential disadvantages may consist of invasiveness, limited indications and reversibility, and side effects, such as increased intraocular pressure, cataract formation, and infection. Verisome (Ramscor, Inc., Menlo Park, CA, USA) is a long-acting intravitreal injectable drug delivery system for different drugs, and it is with TA to treat chronic cystoid macular edema (CME) due to RVO and with ranibizumab to treat nAMD [186,187,188,189].

Ocular implants can provide a sustained and controlled mechanism of drug release over an extended period. They are designed to release medications slowly and maintain therapeutic levels in the eye for a longer duration. This allows for a more consistent therapeutic effect and reduces the need for frequent administration of eyedrops or injections, enhancing patient compliance and convenience. These types of DDSs can enhance drug bioavailability by increasing the residence time of the drug in the eye and in a direct manner at the site of action, ensuring efficient delivery and reducing systemic absorption and consequent potential side effects. They can overcome the challenges associated with short drug half-lives and rapid clearance and can shield drugs from degradation by tear fluid and enzymes, enhancing their stability and prolonging their shelf life. While ocular implants offer several advantages, they also have potential disadvantages. Biodegradable implants generally require a single surgical procedure, while non-biodegradable implants require two surgical procedures under local or general anesthesia, increasing some inherent risks, including infections, bleeding, and damage to surrounding tissues. Additionally, the need for surgery may not be suitable for all patients, especially those who are not surgical candidates or have contraindications. Another disadvantage is irreversibility because some ocular implants, such as sustained-release devices, are designed to remain in the eye for an extended period of time; therefore, they cannot be easily removed or adjusted once implanted. While this may be advantageous for long-term treatment, it can pose challenges if there are complications, adverse reactions, or the need to change the treatment plan. Another limitation is the lack of flexibility due to the fact that, once implanted, it may not be possible to modify the drug or change the medication regimen easily. Like any foreign body, ocular implants carry a risk of complications, including infection, inflammation, implant migration, erosion, or extrusion, which can compromise the effectiveness of the treatment and may require further medical or surgical intervention. In retinal disease, ocular implants have shown promising results in improving visual function, restoring vision, and providing targeted drug delivery to the retina. It is important to note that these routes of administration are still evolving, and their effectiveness may vary depending on the specific retinal condition, patient characteristics, and implant design.

#### 4.3.3. Hydrogels

Hydrogels are well-defined structures composed of a wide variety of hydrophilic monomers connected with crosslinked bonds, which are capable of swelling when placed in water or an aqueous environment [190]. They are highly soft and elastic in nature, have physicochemical similarities with ocular fluids, and are adequate for intraocular use [176]. Hydrogels have been developed to deliver proteins, peptides, and antibodies, as the formation of the hydrogel can occur at ambient temperature conditions. These components are currently used in clinical research utilizing soft contact lenses and foldable intraocular lenses to deliver drugs to the anterior segment and to the posterior segment of the eye and have been considered in the treatment of diabetic retinopathy, age-related macular degeneration, or rhegmatogenous retinal detachment [177,191,192,193].

Several in situ hydrogel systems are prepared with hyaluronic acid, chitosan, poloxamer, hydroxypropyl methylcellulose (HPMC), and polycaprolactone. These components have demonstrated safe use as depot systems in the ocular environment. They can be injected into the vitreous cavity through a small gauge needle, as “in situ”, which then transforms into gel in response to internal and/or external stimuli mediated by pH, temperature, ions, or enzymes [194]. The sol-gel phase transition occurs within seconds to minutes, entrapping, and stabilizing the therapeutic proteins in an aqueous polymeric network [195]. After injection, the hydrogel forms a reservoir, allowing for the continuous release of loaded protein molecules over time [196,197]. Drug release occurs via diffusion-controlled and degradation-controlled processes from the reservoir [198,199].

Hydrogels can play a vital role in the development of nanotechnologies for the delivery of drugs to the eye. Regarding posterior segment delivery, polymeric natural-based hydrogels and synthetic hydrogels are characterized by easy injectability and long residence times, thus potentially promising candidates for ideal vitreous substitutes [200]. It has been demonstrated that polysaccharide crosslinked hydrogels showed sustained release of bevacizumab for three days with a low initial burst, while thermosensitive hydrogels exhibited sustained release of bevacizumab for 18 days [201]. In a recent study, the hyaluronic acid-bearing furan with 4 arm-PEG10K-maleimide (4APM-HAFU) (ratio 1:5) hydrogel formulation was easily injected into the vitreous cavity using a small needle (29G) and showed sustained release of bevacizumab >400 days by a combination of diffusion, swelling, and degradation. A bioassay showed that the released bevacizumab remained bioactive [202].

This hydrogel platform offers high potential for the sustained release of therapeutic antibodies to treat ocular diseases, such as age-related macular degeneration. An injectable antibody-loaded supramolecular nanofiber hydrogel has been created by simply mixing betamethasone phosphate (BetP), a clinical anti-inflammatory drug, anti-VEGF, the gold-standard anti-VEGF drug for AMD treatment, with CaCl_2_. Upon intravitreal injection, such BetP-based hydrogel (BetP-Gel), while enabling a long-term sustained release of anti-VEG, can also scavenge reactive oxygen species to reduce local inflammation and prolong the effective treatment time of conventional anti-VEGF therapy [203]. Overall, various pre-clinical studies on rabbit eye models have shown good biocompatibility of anti-VEGF-loaded hydrogel after intravitreal injection, exhibiting sustained release properties [195,204,205]. These agents may be readily translated into clinical use for AMD treatment with the potential to replace current anti-VEGF therapy.

In vivo investigation with fibrin hydrogels revealed high short-term subretinal biocompatibility of this type of DDS, which is a suitable scaffold for human Embryonic Stem Cell- Derived Retinal Pigment Epithelial Cells (hESC-RPE) transplantation, which could be a new grafting material for tissue engineering RPE cells [206]. A small series of rhegmatogenous retinal detachment models with short-term follow-up provide preliminary evidence to support the favorable biocompatibility and efficacy of the hyaluronic acid (HA) hydrogel as a promising retinal patch for sealing retinal breaks in retinal detachment repair [207]. Moreover, gene delivery using an injectable hydrogel has also been studied as a treatment for retinitis pigmentosa [208,209].

Hydrogels can be combined with microparticles/nanoparticles (NPs) in order to form a hybrid “combined-DDS” for the controlled delivery of therapeutics, especially for localized applications, which can be useful to increase therapeutic efficacy and to limit particle movement in the eye. An injectable hydrogel loaded with retinal-targeted, biodegradable hybrid NPs with a hyaluronic acid coating has been developed to improve in vivo distribution throughout the vitreous and delivery to retinal cells. It has been shown to greatly improve the administration of sensitive therapeutic molecules to the retina by exploiting both the targeting ability and protective effect of NPs while prolonging their release [210]. The use of anti-VEGF-loaded microspheres suspended within an injectable, egener-responsive hydrogel has shown controlled, extended, and bioactive release for approximately 200 days of ranibizumab and aflibercept in vitro. [211]. In a laser-induced CNV model in nonhuman primate models, Kim et al. demonstrated that an aflibercept-loaded microsphere and hydrogel combination system was an effective treatment for up to 6 months post-injection, without adverse events [212]. Recently, a biodegradable microparticle- and nanoparticle-hydrogel DDS was developed to obtain the simultaneous release of aflibercept and dexamethasone. The Combo-DDS has proven to be an effective method for simultaneously releasing dexamethasone and aflibercept for up to six months. This may eliminate the need for separate dosing regimens of anti-VEGF and corticosteroids for patients with wet AMD [213]. The key advantages of hydrogels include the fact that they can provide sustained and controlled release of drugs within intraocular tissues, such as the aqueous humor and vitreous cavity. The gel-like structure of hydrogels allows for the gradual release of drugs, ensuring a sustained therapeutic effect and reducing the frequency of drug administration. Furthermore, certain ocular drugs, particularly biologics, exhibit low stability and/or short half-life when present in the vitreous humor. By encapsulating these biologics within hydrogels, both drug stability and release duration can be improved. In some ocular conditions, such as retinal diseases, monotherapy may not be adequate to achieve optimal therapeutic efficacy, and hydrogels can integrate different drugs into a single platform, simplifying the treatment regimen and improving patient compliance. Therefore, combination therapy has emerged as a crucial strategy for enhancing clinical outcomes through synergistic effects. This class of DDSs can be designed to specifically target the ocular posterior segment and retina. By incorporating drugs into hydrogels, they can be delivered directly to the desired site of action, minimizing systemic exposure, and reducing potential side effects. This targeted drug delivery approach enhances therapeutic efficacy while minimizing off-target effects. Hydrogels can enhance drug penetration through ocular tissues, including the cornea and sclera, to reach the ocular posterior segment and retina because their gel-like nature can promote sustained contact with eye tissues, facilitating better drug permeation and bioavailability in these deep ocular layers. Disadvantages include the fact that hydrogels may have limitations in terms of their loading capacity for drugs. The gel matrix can accommodate a certain amount of drug molecules, and exceeding this limit may lead to reduced gel integrity or compromised release kinetics. This limitation can restrict the use of hydrogels for high-dose drug delivery or when large quantities of drugs need to be administered. Hydrogels, especially those with higher viscosity, may face challenges in effectively penetrating dense ocular tissues, such as the cornea. Their viscosity can impede diffusion and hinder the efficient delivery of drugs to the desired target sites. This limitation may require additional strategies or modifications to enhance the penetration capabilities of hydrogel-based DDSs. While hydrogels can provide sustained drug release, their long-term stability can be a concern because, over time, they may undergo physical or chemical changes, leading to alterations in their drug release profiles. Factors such as degradation, water evaporation, or interactions with ocular fluids can affect the stability and performance of hydrogel-based DDSs. Ensuring long-term stability is essential for maintaining consistent drug release over extended periods.

While hydrogels offer several advantages as DDSs to the ocular posterior segment, further research is still needed to optimize their performance, ensure long-term stability, and address the specific challenges associated with these targeted applications. Nonetheless, hydrogels hold great promise for delivering therapeutics to these critical ocular regions, potentially revolutionizing the treatment of posterior segment and retinal disorders.

#### 4.3.4. Contact Lens

The use of contact lenses (CLs) as routes of administration has been increasingly evaluated in recent years [214]. The idea of using CLs as carriers of active ingredients is a relatively new strategy that is still being developed and improved. There are two main groups of contact lenses depending on the designed material: soft contact lenses made of hydrogel or silicone hydrogel polymers, and rigid gas-permeable contact lenses. Therapeutic contact lenses include polymeric carriers, such as drug-containing polymer nanoparticles and polymeric implants [215]. In recent years, studies have focused on ways to extend the drug residence time and improve the bioavailability of various lens-based DDSs. Surface modifying methods include dip-coating (soaking), diffusion barrier insertion (Vitamin E), incorporation of functional monomers, ligands, and a polymeric matrix, molecular imprinting, cyclodextrin vaccination, incorporation of colloidal, drug-loaded nanoparticles, or other colloidal nanostructured systems, and surface coating by multilayer film deposition of colloidal nanoparticles or ligands [215]. The advantages of using control-released drug systems in the form of contact lenses are the drug dosing regimen, bioavailability, and prolonged residence time of drugs in the eye [216,217]. The use of contact lenses as a reservoir of drugs is a promising treatment system for: chronic eye diseases (such as glaucoma); ocular allergies; controlled release of antimicrobial peptides on the ocular surface; and drug delivery with antiviral, antifungal, anti-inflammatory, and/or immunosuppressive agents [218,219,220,221,222].

A commercially approved silicon hydrogel CL named ACUVUE^®^ OASYS^®^, has been modified to control the delivery of pirfenidone (PFD) to ameliorate corneal inflammation and fibrosis conditions [223]. The same authors used the ACUVUE^®^ Oasys^®^ and the 1-DAY ACUVUE^®^ TruEyeTM lens soaked with vitamin E. These CL have been employed to evaluate the release profiles of ketorolac tromethamine (KT) and flurbiprofen sodium (FS), which have shown a better drug delivery dosage in the treatment of ocular inflammatory conditions [224]. CLs based on methacrylic acid (MAA) loaded with acyclovir (ACV) and valacyclovir (VACV) have been used to treat ocular keratitis caused by the herpes simplex virus (HSV) [221]. Moxifloxacin (MF) and dexamethasone (DM) were loaded on chitosan-, glycerol-, and polyethylene glycol (PEG)-based CLs, which were developed using the solvent casting approach. These CLs showed efficacy in delivering MF and DM in the treatment of postoperative conditions to prevent ocular infections [225]. Silicone hydrogel soft CLs loaded with brinzolamide or latanoprost have also been developed, allowing constant release of the drug and thus representing a good alternative to eyedrops for treating glaucoma [226,227].

Regarding drug delivery to the posterior segment of the eye, Alvarez-Rivera et al. developed CLs suitable for the local prevention/treatment of diabetes-related eye pathologies. The main idea was to incorporate functional groups into the polymer matrix that could reversibly interact with epalrestat, an aldose reductase inhibitor used for the treatment of diabetic neuropathy, promoting drug accumulation and diffusion through the cornea [228]. Moreover, it has been hypothesized that ocular drug flux to the retina can be improved by providing sustained drug delivery directly to the cornea, increasing drug concentrations at the ocular surface, increasing drug residence time at the ocular surface, and using mechanical forces as a permeation enhancer. In vitro and ex vivo experiments have been performed, and the results have shown that these drug-loaded hydrogels may be useful as ocular devices for regulating the posterior release of epalrestat, thus facilitating its accumulation and diffusion across the cornea [229]. Recently, soft hydrogel CLs have been employed to obtain a sustained release of naringenin (NAR), a flavonoid anti-inflammatory and antioxidant substance used for treating posterior eye segment disease, such as age-related macular degeneration [230,231].

A sustained drug-eluting contact lens positioned directly over the cornea has been developed for drug delivery to the eye, including the retina. This contact lens has shown sustained drug delivery to the retina at therapeutic levels using a dexamethasone delivery system (Dex-DS), which consists of a dexamethasone-polymer film encapsulated inside a contact lens. Rabbits wearing Dex-DS achieved retinal drug concentrations that were 200 times greater than those from intensive (hourly) dexamethasone drops. Conversely, Dex-DS demonstrated lower systemic dexamethasone concentrations. In an efficacy study in rabbits, Dex-DS successfully inhibited retinal vascular leakage induced by intravitreal injection of vascular endothelial growth factor (VEGF) [232]. Recently, a drug-loaded contact lens combined with electrodes placed on cadaver rabbit eyes and positioned diametrically opposite and beyond the limbus has been shown to potentially deliver ionic drugs directly to the vitreous. Incorporation of an electric field with multiple electrodes on a single lens can effectively deliver ionic drugs to the posterior region at levels comparable to current methods, with the benefits of being safer and less invasive [233]. CLs offer several advantages over traditional methods of drug administration, providing enhanced effectiveness and patient convenience. This type of route of administration allows the delivery of drugs directly to the ocular posterior segment and retina, enabling targeted drug delivery, minimizing systemic exposure, and reducing potential side effects. Contact lenses can be designed to release drugs continuously over an extended period, ensuring consistent therapeutic concentration at the target site and enhancing treatment efficacy while reducing the frequency of drug administration. This type of route of administration provides a larger surface area for drug absorption compared to traditional delivery methods, such as eyedrops, allowing for better drug penetration into the ocular tissues, enhancing bioavailability, and optimizing the therapeutic effect. Moreover, modern contact lenses are designed to provide excellent comfort and vision, and by incorporating drug delivery capabilities, patients can benefit from both vision correction and therapeutic drug administration simultaneously. This approach eliminates the need for multiple interventions, enhancing patient convenience and acceptance. This type of route of administration is a non-invasive method that reduces the associated risks and complications while offering a less intimidating option for patients. The ease of administration also makes contact lens-based drug delivery suitable for long-term treatment plans. The drug release rate, duration, and dosage can be adjusted based on the specific needs of each patient’s condition. This level of customization allows for personalized treatment strategies and optimized therapeutic outcomes. Contact lenses can accommodate various types of drugs, including small molecules, biologics, nanoparticles, and gene therapies. Nevertheless, there are also some potential disadvantages and challenges that need to be considered. CLs have a limited capacity to carry drugs due to their small size and thickness. This can restrict the amount of drug that can be loaded into the lens, potentially limiting the duration of drug release or requiring frequent lens replacement. The release kinetics of drugs from CLs may vary depending on factors, such as lens material, drug characteristics, and environmental conditions. Achieving a consistent and controlled release profile can be challenging, potentially leading to unpredictable drug concentrations at the target site. Factors such as tear film composition, blinking patterns, and lens movement on the eye can influence drug release and bioavailability. Certain drugs, particularly larger molecules or those with specific formulation requirements, may not be suitable for contact lens-based delivery. This limitation may restrict the versatility of this route of administration in some cases. The compatibility of different drugs with contact lens materials and their ability to maintain stability during storage and wear can limit the range of drugs that can be delivered using this approach. It is important to note that research and technological advancements are continuously addressing these challenges to improve the feasibility and reliability of CLs for ocular posterior segment and retinal therapies. With further innovation and refinement, these disadvantages can potentially be overcome, leading to more effective and patient-friendly ocular drug delivery methods. While the contact lens-based route of administration for the ocular posterior segment and retinal therapies holds tremendous promise, further research and development are still required to overcome challenges related to drug loading, release kinetics, and biocompatibility.

#### 4.3.5. Microneedles

In recent years, due to intense research and advancements in microtechnology, there has been remarkable interest in the development of microneedles (MNs)-based systems as an alternative, non-invasive form for administering drugs to the eye in order to minimize tissue damage, reduce the disruption of membrane continuity, eliminate the risk of pathogen infections, and improve overall safety.

Simultaneously with the development of single-microneedle technologies, research has focused on microneedle systems/patches for ocular drug delivery, recently, and successfully applied to the cornea or sclera [234]. Ocular microneedles are DDSs that show passive delivery of molecules via arrays of solid MNs coated with drug formulations that dissolve a few minutes after insertion. They provide passive diffusion of therapeutics, overcoming the transport barriers of epithelial tissues, eliminating clearance by conjunctival mechanisms, and minimizing retinal damage [79]. The most common microneedle classifications are based on geometry, the material applied to obtain the systems, the method of fabrication, the drug loading technique, and the mode of drug delivery [235].

Regarding the use of MNs as DDSs to the posterior segment of the eye, a lot of research has been conducted recently. Injection into the suprachoroidal space (SCS) represents an alternative method of ocular drug delivery to the posterior segment allowed by MN-based DDSs because it facilitates targeted distribution to affected chorioretinal tissues through the suprachoroidal pathway. This allows potential efficacy benefits, compartmentalization away from unaffected anterior segment tissues, and a high degree of bioavailability. The commercially approved SCS Microinjector^®^ (Clearside Biomedical, Inc., Alpharetta, GA, USA) was specifically designed to provide non-surgical, reliable, and in-office access to the SCS. Indeed, the first and only FDA-approved SC therapy (CLS-TA) is administered via the SCS Microinjector. (Bausch+ Lomb and Clearside Biomedical 2021) Triamcinolone acetonide injectable suspension (Xipere^®^, Bausch + Lomb) was successfully used as a novel formulation optimized for use with the SCS Microinjector^®^ [236]. This presents an opportunity for safe and effective drug delivery for the treatment of uveitic macular edema and, potentially, for broader use with other drugs to treat other ocular diseases that impact chorioretinal tissues, such as age-related macular degeneration, diabetic retinopathy, and choroidal melanoma [237,238].

Roy et al. presented two types of patches for the delivery of triamcinolone acetonide (TA): a microneedle scleral patch (MSP) and a microneedle corneal patch (MCP). Ex vivo experiments performed on porcine eye globe showed that, in comparison to MCP and TA nanosuspension, MSP obtained much greater TA concentrations in the vitreous humor and choroidoretinal complex after 5 min of application [239]. Amer and Chen fabricated PVA hydrogel-based microneedle arrays for the delivery of immunoglobin G1, a model protein resembling bevacizumab, applied in the treatment of age-related macular degeneration (AMD). The in vitro tests showed an extended release of the active compound compared to the rapid release after injection. The authors indicated that the MN-based arrays show a much more uniform drug release profile than the single injections [240]. Wu and co-workers developed nanoparticle-loaded bilayer dissolving microneedle arrays for the sustained delivery of proteins to the posterior ophthalmic segment. The MNs had adequate mechanical stability to puncture the sclera and degrade very quickly, releasing the nanoparticles (NPs) in less than 3 min. The slow disintegration of the NP-forming matrices resulted in the release of the active ingredient in a prolonged manner [241].

Some of the advantages of MNs as DDSs to the ocular posterior segment consist in the fact that they enable precise, controlled, and targeted delivery of drugs, thanks to their minimally invasive nature. MNs create micropores at the level of the sclera/suprachoroidal space which enhance bioavailability and ensure that a higher concentration of the drug reaches the desired site, increasing the therapeutic efficacy while minimizing the required dosage. Compared to traditional ocular DDSs, such as injections, microneedles provide a non-invasive and pain-free alternative method. They can be designed to deliver drugs in a sustained manner, allowing for prolonged therapeutic effects without the need for frequent administration and improving patient compliance with medication regimens. By avoiding the need for preservatives or exposure to harsh conditions, such as high temperatures or pH, microneedles minimize the risk of drug degradation and ensure that the therapeutic agent reaches the ocular posterior segment in its active form. MNs also have some potential disadvantages that should be considered. Their fabrication can be technically complex and challenging. Achieving the desired dimensions, needle strength, and sharpness while ensuring biocompatibility and reproducibility can be demanding. Developing scalable manufacturing processes for commercial production is an ongoing area of research. Although MNs are designed to be minimally invasive, there is still a risk of injury during the insertion process. Their small size restricts the amount of drugs that can be loaded onto or within them. This limitation may pose a challenge for drugs that require higher doses or those with low solubility. MNs are often made from materials that may have limited stability, especially in terms of long-term storage. Factors such as degradation, moisture absorption, or changes in mechanical properties over time can impact their performance and effectiveness. It is important to note that, while these disadvantages exist, ongoing research and development efforts are focused on addressing these challenges and improving the performance and safety of MN-based ocular DDSs.

Overall, MNs can be considered a minimally invasive compromise between topical formulations, which are acceptable but reveal poor effectiveness, and direct injections, which are more effective but invasive. With this novel approach, the drug can be delivered to the target site with good precision and minimized the risk of tissue damage, pain, and infection [234,242].

## 5. Conclusions and Future Perspectives

**Figure 2:**
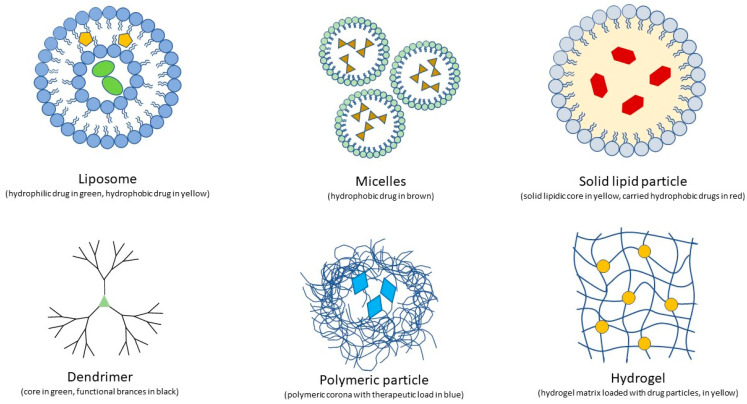
Material used in Drug Delivery Systems (DDSs).

The development of innovative DDSs to increase the passage of therapeutic molecules across the ocular barriers to the posterior ocular segment, with minimally invasive, safe, and affordable technologies, is a rapidly growing research field. Figure 2 shows the different materials used in the current DDSs, while Table 1 lists the characteristics, pros, and cons of each. Various alternatives to ordinary intravitreal injection have been proposed to overcome the clinical and logistic burdens for the patient and for the healthcare providers that are intrinsic to this type of treatment and the chronic nature of most posterior segment disorders. Nanotechnologies, alternative drug penetration enhancers, and various types of in situ devices for sustained drug release have been extensively tested, many, for the moment, only on animal models, in vitro, or with limited numbers and indications on humans. They seem capable of delivering targeted retinal treatments with promising results, but more in vivo studies are required to confirm these experimental results. The addition of mucoadhesive polymers and aggregation with micro/nanomolecular carriers have been shown to enhance drug bioavailability and promote the prolonged release of therapeutic concentrations. Nanoparticles in general have the advantages of small size, considerable surface area, and high mucoadhesivity, whereas the particles’ stability and loading capability is not always optimal. For these reasons, NPs have often been integrated with more complex polymeric structures, such as gel, to integrate the controlled delivery of the first with the higher stability and prolonged retention time of the second. Nevertheless, several aspects of the drug penetration mechanism are still to be clarified, as if it may trigger toxic reactions or harmful alterations to the ocular barriers [121].

Apart from nanotechnologies, other systems have been developed and studied to enhance drug delivery. CPPs, for example, are characterized by a general lack of cell and tissue specificity. Although TAT and penetrates were seen to somehow accumulate in the retina after topical administration, the use of specific CPPs to target different eye structures demands further investigation since the available data derive from studies using different in vivo models and conditions. In vivo experiments are generally conducted on rats or mice. This is often a necessary choice, and differences between murine and human eyes in relevant aspects, including ocular volumes and delivery path lengths, must be considered when interpreting the results of this type of study. Furthermore, evidence of ocular toxicity and uptake of CPPs is collected with experiments on isolated human conjunctival/corneal/RPE cell lines. Although these in vitro results are considered more replicable and less susceptible to effects derived from species-dependent differences, some drawbacks remain. The first difficulty derives from the differences in CPP behavior in different cell lines. For example, it is known that non-adherent cell lines show a higher permeability toward CPPs [243]. A second issue concerns the prediction of the results obtained in vitro when translated in vivo. In the latter set-up, not only pharmacokinetic factors, such as lacrimal wash-out and blood and lymphatic absorption, but also a different CPP interaction with cells integrated in an organized tissue and organism could make the difference. For instance, a reduction in the cellular uptake of peptides for ocular delivery was observed after incubation with proteoglycans, which are naturally present on the cellular surface and intercellular spaces in vivo [138].

Iontophoresis seems to offer a potential route to enhance the transscleral-transconjunctival absorption of topical drugs directed to the vitreous and retina. Again, preclinical studies have been conducted on animals, and some concerns have emerged regarding safety due to the exposure to prolonged electric current and feasibility in terms of logistic and technological costs, especially in comparison to other topical treatments [122].

Different types of implants and inserts have been proposed to grant the controlled and sustained release of ocular drugs, reducing the number of drug administrations, with benefits on the patient comfort and compliance, and reducing the number of necessary accesses to the health care providers for treatment and monitoring. These devices, however, have some limitations. The positioning and size of the implant must be considered to avoid vision obscuration. To this extent, injection of particles below 300 nm is recommended. Light scattering and other vision disturbances can occur because of the aggregation of particles after injection into the vitreous. Systems allowing the sustained release of drugs from nanoparticles may thus offer an advantage. Even with modern technology, it is still rather challenging to load a sufficient amount of drug into small particles, which need to be released for at least a few weeks after a single administration. This factor represents the true bottleneck in developing a successful intravitreal treatment of this kind. For these reasons, sustained efficacy of action in the posterior segment might be achieved by subconjunctival injection of products with slow choroidal clearance or by using a formulation that provides gradually releasing drugs in the suprachoroidal space. Overall, research in this sector only produced a few implants now available for clinical use, with some of the most promising solutions, such as nanotechnologies, not really translating the good results obtained in preclinical studies into clinical trials. Furthermore, according to some authors, this might also be due to the scarce investment of nanotechnologies in the development of neurological and ocular treatments, with more tangible efforts made to develop oncological and imaging agents [123].

Despite these considerations, the existence of different types of implants and polymeric materials, with many of them already approved for clinical use, and the continuous development of new drugs to treat diffuse pathologies, such as AMD and RD, makes efficiency in drug delivery a highly dynamic field with interesting and crucial applications. Therapeutic efficacy is certainly the primary objective of any DDS; however, a patient’s comfort is also crucial since it considerably affects compliance with treatment, which is also a key factor, especially when it comes to ocular treatments. More trials on existing technologies and the development of new strategies will allow for advancements in therapeutic efficiency with reduced side effects and better compliance in treating pathologies of the posterior ocular segment.

## Figures and Tables

**Table 1:** Strategies for drug delivery to the ocular posterior segment.

		Brief Description	Developmental Stage	Advantages	Disadvantages
Nanomedicine	Liposomes	Membrane-like vesicular structures carrying drugs across ocular barriers, e.g., Visudyne (Bausch&Lomb) to deliver verteporfin to the retina in nAMD Dexamethasone and tacrolimus also experimented with using liposomes to treat vitreoretinal inflammation and macular edema	Approved (Visudyne)	Good biocompatibility and biodegradability Low toxicity Better drug-loading capacity compared to other lipidic nano vesicles Effective protection of carried molecules	Unstable preparation (aggregation during transportation and storage with loss of transparency) Need for stabilizing excipients Questionable physicochemical properties Possibile inflammatory and hypersensitivity reactions
	Nanomicelles	Amphiphilic monolayered vesicles carrying drugs. Topical preparations able to vehicle drugs to the retina via the conjunctival-scleral route in a safe and non-invasive way. Intravitreal administration is more invasive but may be useful to obtain sustained drug release (e.g., intravitreal pazopanib-loaded nanotubes)	Pre-clinical	Small size allows for sub-cellular targeting Water-insoluble drug loading capacity Simple and stable structure with spontaneous formation and low aggregation tendency Long circulation time (by-pass hepatic macrophages)	Considerably susceptible to environmental changes: easy content precipitation Hydrophobic load only
	Nanospheres and nanocapsules	Self-assembling polymeric nanospheres and nanocapsules	Pre-clinical	Easy formation: self-assembling Uniform drug-load distribution: less inflammatory	Low drug-loading efficacy Need encapsulation in polymeric delivery systems
	Solid lipids	Solid lipid core stabilized by surfactants. Stable, extremely resistant (autoclave), and economic	Pre-clinical	Resisting (autoclave) and long standing structure Biodegradable Inexpensive production	Susceptible to content expulsion during storage Hydrophobic load only
	Dendrimers	Ramified polymers consisting in a core with functional groups on its branches Intravitreal steroids, subconjunctival antiblastic, and topical antiglaucomatous molecules have been tested on animal models	Pre-clinical	Good targeting capability Versatile structure Advantageous slow clearance in target tissues	Potentially cytotoxic
	Organic nanopolymers	Chemical compounds acting as carrier for retinal drugs including dexamethasone and anti-VEGF, enhancing their bioavailability and targeting and prolonging their release	Pre-clinical	Biocompatible, stable, biodegradable materials with well-documented safety profile in humans Easily targetable compounds Can be combined with different nanoparticles Blood–retina barrier crossing documented in vivo	Corneal and vitreous opacification and inflammation reported with PVP PLGA presents weak protein stability, inefficient drug loading, abrupt release profile Crystallinity alteration induced by copolymers.
	Chitosan	Highly diffuse polysaccharide used for decades in pharmaceutics. Versatile penetration enhancer employed as a coating or component of different drug-loaded particles and drug delivery systems.	Approved products containing chitosan	Natural molecule with no cititoxicity High biocompatibility and biodegradability Mucoadhesive nature	
	Metals and other inorganic materials	Metallic nanoparticles with intrinsic therapeutic properties. Magnetism can be used to direct ab-externo metallic nanocarriers to target tissues (e.g., helical vectors containing iron and nickel)	Pre-clinical	Antiangiogenic activity (silver, gold, silicate) Inflammatory effects (gold) Ability to reduce vascular permeability (silver) Antiapoptotic and antioxidant action (nanoceria)	Poor biodegradability and clearance Potentially toxic and cell damage (photoreceptors) Compromise BRB integrity
	Encapsulated cells	Implantable polymeric scaffold containing human RPE cells secreting growth factors	Pre-clinical	Sustained secretion from living cells Potentially applicable to multiple types of cells and diseases	Invasive implantation Metallic device, not biodegradable
Topical drugs penetration enhancers	Cell-penetrating peptides	Short chains of peptides trespassing membranes. Studied on animal models of choroidal neovascularization, retinal neovascularization and oxygen-induced retinopathy	Pre-clinical	High targetability No cytotoxicity Non-corneal pathways involved	
	Cyclodextrins	Oligosaccharides with a truncated cone-shape, more lipophilic centrally and a more hydrophilic on the outer surface, which can be used to aggregate with and augment the topical absorption of hydrophobic drugs	P2	Biodegradable molecules Good protection and stabilization of the carried drug Improved solubility, bioadhesion, and permeation of the treatment Targeted and sustained drug delivery	Poor hydrosolubility and affinity with hydrophobic drugs, requiring addition of moieties
	Benzalkonium chloride	Enhancing penetration by breaking down tight junction of corneal epithelia.	Pre-clinical as enhancer for posterior segment disease treatment	Widely used and approved molecule	Intrinsic damage to ocular barriers
	Iontophoresis	Application of low-amplitude electrical current to vehicle drugs across the conjunctival-scleral barrier	P1 (Visulex) Launched (EyeGate II)	Anterior ocular surface sparing technique (transconjunctival-transscleral pathway)	Expensive technology Dedicated device and drug formulation required Concerns about skin exposure to prolonged electric current
Sustained Drug-release Systems	Ocular inserts	Sterile, non-implantable, thin, multi-layered routes of administration with solid or semisolid consistency	Pre-clinical	Increased contact time/bioavailability Long-lasting, controlled, and precise release Minimal systemic absorption Reduced administration frequency	Patient non-compliance with foreign body sensation Difficulty in self-insertion or removal Potential accidental loss Potential obstacle to vision due to movement around the eye
	Non-biodegradable Ocular Implants	Scleral and intrascleral (disc) implants and intravitreal implants (encapsulated cells) which do not experience change in structure and are made up of ethylene vinyl acetate, polyvinyl alcohol, polysulfone capillary fiber	Approved	Prolonged drug release Improved drug bioavailability Enhanced therapeutic efficacy Reduced dosing frequency	Surgical procedure Non-biodegradable Irreversibility Limited drug flexibility Implant-related complications
	Biodegradable Ocular Implants	Injectable microparticles, intravitreal implant (injectable rod), intra-scleral and epi-scleral implant (disc, plug) which degrade and disintegrate over time. They are made up of polylactic acid, polyglycolic acid, PLGA, or polycaprolactones	Approved		
	Hydrogels	Three-dimensional network structures of crosslinked hydrophilic monomers which can deliver drugs via multiple administration routes such as topical administration, intracameral injection and intravitreal injection	Pre-clinical	Controlled and sustained drug release Site-specific drug delivery Enhanced drug stability and penetration Possible combination therapy	Limited drug-loading capacity Difficulty penetrating dense ocular tissues Lack of long-term stability
	Contact lens	Contact lenses act as a drug-reservoir, incorporating functional monomers or nanoparticles in a polymeric matrix	Pre-clinical or approved	Targeted delivery Prolonged drug release Improved bioavailability Enhanced patient comfort Non-invasive approach	Limited drug capacity Variable drug release Individual variability Limited drug selection
	Microneedles	Drug-loaded arrays of microneedles, inserted in sclera/suprachoroidal space	Pre-clinical or approved	Enhanced targeted delivery Improved drug bioavailability Non-invasive method Sustained release Preservation of drug integrity	Technical challenges Possible risk of ocular damage and infection Limited drug payload Limited stability and shelf life

## Data Availability

Not applicable.
